# Autoimmune conditions are associated with perioperative thrombotic complications in liver transplant recipients: A UNOS database analysis

**DOI:** 10.1186/s12871-016-0192-3

**Published:** 2016-05-21

**Authors:** Dmitri Bezinover, Khaled Iskandarani, Vernon Chinchilli, Patrick McQuillan, Fuat Saner, Zakiyah Kadry, Thomas R. Riley, Piotr K. Janicki

**Affiliations:** 1 Department of Anesthesiology, Penn State College of Medicine/Penn State Hershey Medical Center, 500 University Drive, Hershey, PA 17033 USA; 2 Department of Public Health Sciences, Penn State College of Medicine/Penn State Hershey Medical Center, 90 Hope Drive, Hershey, PA 17033 USA; 3 Department of General, Visceral- and Transplant Surgery/Essen University Medical Center, Hufelandstr. 55, Essen, 45147 Germany; 4 Department of Surgery, Penn State College of Medicine/Penn State Hershey Medical Center, 500 University Dr, Hershey, PA 17033 USA; 5 Department of Gastroenterology, Penn State College of Medicine/Penn State Hershey Medical Center, 500 University Drive, Hershey, PA 17033 USA

**Keywords:** Liver transplantation, Portal vein thrombosis, Hepatic artery thrombosis, Autoimmune and oncologic conditions, Antithrombotic prophylaxis

## Abstract

**Background:**

End stage liver disease (ESLD) is associated with significant thrombotic complications. In this study, we attempted to determine if patients with ESLD, due to oncologic or autoimmune diseases, are susceptible to thrombosis to a greater extent than patients with ESLD due to other causes.

**Methods:**

In this retrospective study, we analyzed the UNOS database to determine the incidence of thrombotic complications in orthotopic liver transplant (OLT) recipients with autoimmune and oncologic conditions.

Between 2000 and 2012, 65,646 OLTs were performed. We found 4,247 cases of preoperative portal vein thrombosis (PVT) and 1,233 cases of postoperative vascular thrombosis (VT) leading to graft failure.

**Results:**

Statistical evaluation demonstrated that patients with either hepatocellular carcinoma (HCC) or autoimmune hepatitis (AIC) had a higher incidence of PVT (*p* = 0.05 and 0.03 respectively). Patients with primary biliary cirrhosis (PBC), primary sclerosing cholangitis (PSC) *and* AIC had a higher incidence of postoperative VT associated with graft failure (*p* < 0.0001, *p* < 0.0001, *p* = 0.05 respectively). Patients with preoperative PVT had a higher incidence of postoperative VT (*p* < 0.0001). Multivariable logistic regression demonstrated that patients with AIC, and BMI ≥40, having had a transjugular intrahepatic portosystemic shunt, and those with diabetes mellitus were more likely to have preoperative PVT: odds ratio (OR)(1.36, 1.19, 1.78, 1.22 respectively). Patients with PSC, PBC, AIC, BMI ≤18, or with a preoperative PVT were more likely to have a postoperative VT: OR (1.93, 2.09, 1.64, 1.60, and 2.01, respectively).

**Conclusion:**

Despite the limited number of variables available in the UNOS database potentially related to thrombotic complications, this analysis demonstrates a clear association between autoimmune causes of ESLD and perioperative thrombotic complications. Perioperative management of patients at risk should include strategies to reduce the potential for these complications.

## Background

Orthotopic liver transplantation (OLT) for patients with end-stage liver disease (ESLD) has become a standardized procedure with an acceptable 5-year survival in the majority of established liver transplantation programs in the world. Despite significant improvements in all aspects of patient management, OLT is associated with a number of complications affecting patient outcome. One of the most significant complications is a perioperative vascular thrombotic event (PVTE). PVTE is associated with an increased mortality and morbidity in each stage of perioperative management. PVTE is not infrequently the cause of postoperative graft failure [1–3].

In this study we analyzed a number of factors potentially associated with PVTE.

Retrospective analysis of United Network for Organ Sharing (UNOS) data was performed to evaluate the effect of oncologic and autoimmune diseases on the incidence of perioperative vascular thrombotic complications in patients undergoing OLT. An association between oncologic and autoimmune conditions and an increased risk of thrombosis in general is well known [4–7]. Combining these disorders with ESLD potentially enhances the likelihood of a patient developing a hypercoagulable state. In addition, statistical analysis was performed to determine the role of other factors in the development of pre- or postoperative thrombotic events.

## Methods

The study was approved by the Institutional Review Board at the Penn State Hershey Medical Center.

In this retrospective study, we analyzed the incidence of thrombotic complications in adult deceased donor LT recipients using the UNOS database. Overall, 119,663 liver transplantations performed in the US between 1993 and 2012 were available for review. Due to incomplete data, only OLTs performed between 2000 and 2012 were evaluated in the study. Patients younger than 15 years old and patients who received living donor liver transplantation were excluded from data collection.

We evaluated the association between PVTE and conditions known to be associated with a hypercoagulable state:

1. Hepatocellular Carcinoma (HCC)
2. Primary Biliary Cirrhosis (PBC)
3. Primary Sclerosing Cholangitis (PSC)
4. Autoimmune Cirrhosis (AIC)

Thrombotic complications identified in the database included:

1. Preoperative portal vein thrombosis (PVT)
2. Postoperative vascular thrombosis (VT) as a cause of graft failure (type of vascular complication was not defined in the database)

Analysis included:

1. The association between oncologic and autoimmune conditions and preoperative PVT.
2. The association between oncologic and autoimmune conditions and postoperative VT.
3. The association between pre- and postoperative thrombotic events

Additionally, multivariable logistic regression analysis of PVTE predictive factors was performed**.** The variables used for multivariable logistic regression were chosen based on expert opinion and previously published data describing factors associated with thrombosis in this patient population. Only a limited number of variables were included in the analysis due to the limited availably of data in the UNOS database.

## Statistics

SAS version 9.3 (SAS Institute, Cary, NC) was used to conduct all the data management and analyses. The Pearson Chi-square test was used to test for differences in incidence proportions of both thrombosis outcomes across discrete clinical and demographic characteristics. A two-tailed t-test was used to test for differences in outcomes across continuously distributed recipient characteristics.

Two multivariable logistic regression models for each of the outcomes were developed. The initial selection of variables to include in the models was performed by expert clinician opinion and a review of the literature for factors associated with thrombosis during liver transplantation. Using a step-wise selection method with a probability threshold cutoff of 0.05, the following variables were dropped from the models: creatinine, international ratio of prothrombin, bilirubin, indicator whether patient had dialysis within two weeks prior to transplant (MELD score components), warm and cold ischemia times, donor’s age, ABO mismatch, HCC, HCC and cirrhosis, and other cirrhosis. PSC and PBC were only dropped from the portal vein thrombosis model. Both models controlled for regional and temporal variations by including time and region indicator variables. The Bayes Information Criterion was used to select the final models.

## Results

We evaluated 62,441 OLTs performed between 2000 and 2012. Statistical analysis was performed to determine any association between PVTE and the following autoimmune and oncologic conditions: a) HCC (*n* = 1,956), b) HCC and cirrhosis (*n* = 3,205) (HCC and HCC with cirrhosis were separately listed in the database), c) PBC (*n* = 2,103), d) AIC (*n* = 1,746), and e) PSC (*n* = 3,030). UNOS data related to thrombotic complications for this time interval included: 1) preoperative PVT at the time of transplant (*n* = 4,247), and 2) postoperative VT leading to graft failure (*n* = 1,233). Demographics and incidence of thrombotic events are demonstrated in Table 1.

**Table 1:** Demographic and clinical characteristics of the UNOS liver transplantation sample for the time interval 2000-2012 by portal vein Thrombosis and graft failure ascribed to vascular thrombosis

	Preoperative Portal Vein Thrombosis			Postoperative Vascular Thrombosis		
Demographic Characteristics	Yes (*N* = 4,247)	No (*N* = 61,399)	*P*-value	Yes (*N* = 1,233)	No (*N* = 6,824)	*P*-value
Age (year)	54.1	52.4	<0.0001*	49.8	50.5	0.04*
Sex (Male) (%)	2,952 (69.5 %)	40,893 (66.6 %)	<0.0001*	834 (67.6 %)	4,559 (66.8 %)	0.58
BMI						
Underweight (%) (BMI < 18.5 kg/m^2^)	104 (2.5 %)	1,498 (2.4 %)	0.97	51 (4.1 %)	173 (2.5 %)	0.002*
Overweight (%) (≥25 kg/m^2^)	1,418 (33.4 %)	21,605 (35.2 %)	0.018*	401 (32.5 %)	2,454 (36.0 %)	0.02*
Obese (%) (BMI ≥ 30 kg/m^2^)	1,549 (36.5 %)	20,003 (32.6 %)	<0.0001*	392 (31.8 %)	2,184 (32 %)	0.90
Clinical Factors						
TIPSS (%) at the time of LTX	502 (11.8 %)	5,156 (8.4 %)	<0.0001*	93 (7.5 %)	510 (7.5 %)	0.06
Portal Hypertensive Bleeding (%) at time of LTX	73 (8.8 %)	636 (4.4 %)	<0.0001*	9 (2.8 %)	129 (6.7 %)	0.02*
Portal Vein Thrombosis (%) at the time of LTX	--	--	--	136 (11.0 %)	373 (5.4 %)	<0.0001*
Diabetes mellitus (%)	925 (23.9 %)	8,586 (15.5 %)	<0.0001*	140 (12.7 %)	700 (11.7 %)	0.39
Autoimmune Cirrhosis (%)	135 (3.2 %)	1,607 (2.6 %)	0.03*	42 (3.8 %)	165 (2.7 %)	0.05*
Primary Sclerosing Cholangitis (%)	--	--	--	97 (7.9 %)	288 (4.2 %)	<0.0001*
Primary Biliary Cirrhosis (%)	--	--	--	48 (4.3 %)	158 (2.6 %)	0.002*
Hepatocellular carcinoma (%)	146 (3.8 %)	1,809 (3.2 %)	0.05*	23 (2.0 %)	258 (2.5 %)	0.33

*p-values indicate a statistically significant difference at α = 0.05

*LTX* Liver transplantation

A significantly higher incidence of preoperative PVT was found in patients with HCC and AIC in comparison to other patient populations (*p* = 0.05 and *p* = 0.03 respectively). A high incidence of postoperative VT was found in patients with PBC, PSC (*p* < 0.0001), and AIC (*p* = 0.05). In addition, patients having had a preoperative PVT had a significantly higher incidence of postoperative VT (*p* < 0.0001).

Multivariable logistic regression analysis demonstrated a number of significant associations (Table 2). Patients with AIC, obese patients with a BMI above 40, patients with diabetes mellitus (DM), and those having had a TIPS, were more likely to have had a preoperative PVT (OR 1.36, 1.19, 1.22 and 1.78 respectively).

**Table 2:** Multivariable logistic regression. UNOS liver transplantation sample for the time interval 2000-2012 by portal vein thrombosis and graft failure ascribed to vascular thrombosis

	Preoperative Portal Vein Thrombosis			Postoperative Vascular Thrombosis		
	OR	95 % CI	P-value	OR	95 % CI	P-value
Autoimmune Cirrhosis	1.36*	1.10—1.69	0.005*	1.64*	1.12—2.40	0.01*
Primary Biliary Cirrhosis	**--**	**--**	**--**	2.09*	1.47—2.98	<0.0001*
Primary Sclerosing Cholangitis	**--**	**--**	**--**	1.93*	1.48—2.51	<0.0001*
Portal Vein Thrombosis at the time of LTX	**--**	**--**	--	2.01*	1.58—2.55	<0.0001*
Underweight (BMI < 18.5 kg/m^2^)	1.04	0.79—1.38	0.76	1.60*	1.07—2.40	0.02*
Obese (BMI ≥ 40 kg/m^2^)	1.19*	1.08—1.31	0.0005*	1.00	0.83—1.19	1.00
Overweight (≥25 kg/m^2^)	1.05	0.96—.16	0.31	0.99	0.83—1.17	0.88
Normal Weight (18.5 ≥ BMI < 25)	**--**	**--**	**--**	**--**	**--**	**--**
Sex (Male)	1.09*	1.00—1.18	0.04*	1.00	0.86—1.17	0.97
Age (Year)	1.01*	1.01—1.02	<0.0001*	0.99	0.99—1.00	0.04
TIPSS at the time of LTX	1.78*	1.53—2.06	<0.0001*	0.82	0.57—1.16	0.25
Diabetes mellitus	1.22*	1.01—1.26	<0.0001*	**--**	**--**	**--**

*Statistically significant at α = 0.05

*LTX* Liver transplantation

Note: This Model included the regions and year indicator variables to control for observed regional and temporal variations, the estimates are omitted for brevity

Postoperative VT were highly associated with AIC, PSC, and PBC (OR 1.64, 1.93, and 2.09 respectively). Patients having had a preoperative PVT were more likely to have had a postoperative VT (OR 2.01). In addition, patients with a BMI ≤18 also had an increased risk of postoperative VT (OR 1.6).

Considering the very strong association between TIPS and PVT, additional bivariate analysis was performed on the data for this patient population.

There were 5,658 patients having had a TIPS at the time of transplant. Overall, there was a significantly higher incidence of patients having had a TIPS who had a PVT (8.48 % vs. 4.05 %, *p* < 0.0001). Stratifying data for patients having had a TIPS with respect to the cause of liver failure demonstrated that the combination of TIPS with AIC had a higher incidence of PVT as compared to patients without TIPS and AIC (19.4 % vs. 8.4 %, *p* < 0.0001).

## Discussion

Our study demonstrates that autoimmune conditions are strongly associated with PVTE. A possible association between oncologic conditions and PVT was also found. In addition, PVT *at the time of transplantation* was a strong predictive factor for the development of postoperative VT.

Chronic liver disease is associated with thrombotic complications throughout the perioperative period with the overall incidence of venous thrombosis varying between 0.5 and 6.3 % [8–10]. Hepatic artery thrombosis specifically, was found in 3–9 % cases and usually occurred after transplantation. It is, however, associated with significant morbidity resulting in up to 53 % of all post-transplant graft losses [2].

Another major factor contributing to perioperative morbidity is PVT. The prevalence of PVT in liver transplant candidates prior to transplantation is 8–25 % [11–13]. Post-transplant PVT occurs in 2–4 % of patients and is associated with significant postoperative mortality [1, 14].

Although the cause of PVTE is likely multifactorial, hypercoagulability associated with ESLD is frequently underestimated as a contributing factor. It has been demonstrated that despite significant decreases in the concentration of both coagulation and anticoagulation factors, patients with ESLD have a compensatory increased concentration of liver-independent factors such as Factor VIII, von Willibrand factor (VWF), and Plasminogen Activator Inhibitor-1 (PAI-1) [15–19]. Both the plasma level and activity of VWF and PAI-1 remain increased up to 10 days after transplantation [14, 20, 21]. It has also been demonstrated that the concentration of ADAMTS13 (protein responsible for splitting VWF) is significantly decreased during and after OLT [21]. Taking into consideration that modern platelet function tests have failed to identify platelet dysfunction in patients with ESLD [22], a VWF/ADAMTS13 imbalance might be responsible for the hypercoagulability often seen in this patient population [14, 21]. Other factors, such as increased levels of thrombin [23], lipopolysaccharides and tissue factor (TF), as well as resistance to thrombomodulin [24, 25] also contribute to the hypercoagulable state seen in patients with ESLD. This hypercoagulability is enhanced if the ESLD is caused by oncologic or autoimmune diseases.

Our study demonstrates an increased incidence of preoperative PVT in patients with HCC. Previous investigations found the incidence of PVT in association with HCC to be between 20 and 65 % [4, 5]. PVT after transplantation in patients with HCC is also associated with an increased mortality (OR 2.05, *p* = 0.004) [26, 27]. It has been suggested that increased TF expression might be responsible for both PVT and systemic venous thrombosis [26, 28, 29].

Our investigation demonstrated a significant association between PBC, PSC, AIC, and postoperative VT. All these conditions are autoimmune and include characteristics contributing to hypercoagulability which include: chronic inflammation (release of cytokines, expression of tissue factors, endothelial dysfunction, inhibition of protein C), increased levels of PAI-1 and antiphospholipid antibodies, as well as a number of other factors such as elevated levels of fibrinogen and TF [6, 7, 30–33].

It has been reported that preoperative PVT might be a risk factor for recurrent postoperative thromboses resulting in an increased risk of re-transplantation [25, 34]. We also found that preoperative PVT was associated with postoperative thrombotic complications. It has been suggested that an underlying hypercoagulability related to rebalanced hemostasis might be responsible for this phenomena [14].

Our study demonstrated a significant association between DM and PVT. Previous investigations have shown an increased risk of venous thrombosis in patients with DM [35, 36]. This finding is likely related to an increased concentration of circulating microparticles, as well as elevated thrombin and estrogen levels seen in patients with DM [37, 38]. Taking into consideration that patients with ESLD have elevated estrogen levels at baseline, the increase seen in patients with ESLD and DM might contribute to the development of PVTE in this population.

There were a number of other important findings in our investigation. Patients extremely over- or underweight had a higher incidence of PVTE. The relationship between obesity and thrombotic complications is well known [39–45]. The association between thromboses and being underweight has not been previously described and is most likely underappreciated. Pelletier, et al., found that patients with a BMI ≤20 had a significantly higher risk of death while on the transplant waiting list (OR = 1.61, *p* < 0.0001) [46]. The cause of this phenomenon is not completely clear, but malnutrition-related mechanisms, such as increased platelet aggregation with a concomitant elevation in thromboxane A [47], and decreased vitamin B6 levels resulting in hyperhomocysteinemia, might play an important role [48, 49]. The problems related specifically to being underweight and having a liver transplant should be evaluated more extensively in the future.

Recent publications have demonstrated that postoperative thrombotic complications can be successfully prevented using antithrombotic medications [50, 51]. Villa, et al., demonstrated the effectiveness of enoxaparin in preventing PVT in patients with advanced cirrhosis [50]. It has been shown that either aspirin administration [51] or an infusion of low dose heparin [52] was effective in the prevention of postoperative HAT. In their study, no bleeding complications were observed.

Considering that autoimmune-related ESLD is associated with up to a twofold increased risk of perioperative thrombotic complications, prophylaxis and treatment of PVTE in patients *at risk* must be considered.

The relationship between TIPS and PVT has been previously demonstrated [53]. In our study, we also demonstrated this association. TIPS placement for PVT treatment was previously described in a small patient population as an experimental procedure [54]. More recently, trans-splenic portal vein recanalization in conjunction with TIPS placement has been reported [55]. With the development of this new TIPS procedure to *treat* PVT, some of the TIPS placements listed in the database may have been inadvertently included in our analysis of TIPS as the *cause* of PVT. It is difficult to estimate how this may have affected our statistical results. Considering that TIPS for PVT treatment was established after 2012 and until now not performed routinely in US centers, we expect the statistical impact to be negligible. The association between TIPS and thrombotic complications remains incompletely understood and needs further evaluation.

Our analysis of the UNOS database has a number of limitations.

Considering the retrospective character of this evaluation, prospective randomized trials are necessary to establish definitive recommendations for routine perioperative antithrombotic prophylaxis for ESLD patients with autoimmune conditions.

While performing this study, we found a significant limitation in the structure of the UNOS database. The number of potentially confounding factors, such as perioperative transfusion of blood products and coagulation factors, the use of antifibrinolytics, intraoperative blood loss, and type of surgical technique were not listed in the database and could not be included in our statistical analysis. Many transplant centers have independently established protocols for perioperative anticoagulation. This information was also not available for analysis. Although these factors which were not included in the data base may confound, to a limited extent, the effects of the predictors included in our model, they would not change the direction of our modeled effects nor impact the validity of our results. Considering the significant statistical power of this analysis and the large number of available variables known to be related to thrombotic complications, we are confident our results demonstrate a true clinical association between autoimmune conditions and PVTE’s.

The type and timing of postoperative VT was not defined in the database. Taking into consideration that only VT’s resulting in graft failure were listed, the vast majority of postoperative thromboses were likely related to HAT as previously described [2]. Problems with the hepatic artery anastomosis are a common cause of HAT especially in the pediatric population [56]. Considering the significant statistical power of our study, we assumed a normal distribution of technical problems in all analyzed patients and excluded pediatric patients. Taking into account these considerations, our results demonstrate an increased postoperative VT risk in patients with autoimmune conditions. More detailed risk stratification, including the type of postoperative thrombotic complication, may be obtained from future research.

Another limitation of the database is that only 2 types of thrombotic complications were listed. This could result in the appearance of a disproportional incidence of pre- and postoperative thrombosis in patients with PBC and PSC. These autoimmune conditions can potentially be associated with other types of thromboses such as deep venous, pulmonary, and others not listed.

The limited types of thrombotic events listed might account for our finding that HCC was not associated with thrombotic events. A significant number of patients with HCC undergo transarterial chemoembolization (TACE). This potentially results in arterial damage with subsequent thrombosis [57]. In our investigation, however, we were not able to demonstrate a relationship between HCC and postoperative VT despite significant patient numbers.

## Conclusion

Identifying factors contributing to thrombotic events as well as the prevention of PVTE is of high importance. In our study, we have demonstrated that autoimmune disorders, such as PBC, PSC and AIC, can lead to an increased incidence of PVTE. Preoperative PVT is associated with postoperative thrombotic complications that are likely related to an underlying hypercoagulable state. Based on our results, postoperative antithrombotic prophylaxis for patients with autoimmune disorders undergoing OLT should be carefully considered.

## Ethics approval and consent to participate

This study was approved by the Institutional Review Board at the Penn State Hershey Medical Center (STUDY00000014). The Review Board waived informed consent due to the retrospective design of the study and anonymized data collection.

## Availability of data and materials

United Network for organ Sharing database was used for this analysis. Data can be requested at: https://optn.transplant.hrsa.gov/converge/data/about/OPTNDatabase.asp.

## Abbreviations

AIC: autoimmune cirrhosis
CI: confidence interval
DM: diabetes mellitus
ESLD: end-stage liver disease
HAT: hepatic artery thrombosis
HCC: hepatocellular carcinoma
OLT: orthotopic liver transplantation
OR: odds ratio
PAI-1: plasminogen activator inhibitor-1
PBC: primary biliary cirrhosis
PSC: primary sclerosing cholangitis
PVT: portal vein thrombosis
PVTE: perioperative vascular thrombotic events
TF: tissue factor
TIPS: transjugular intrahepatic portosystemic shunt
UNOS: United Network for Organ Sharing
VT: vascular thrombosis
VWF: von Willibrand factor
